# Frequencies of interleukin-6, GST and progesterone receptor gene polymorphisms in postmenopausal women with low bone mineral density: Frequência do polimorfismo da *interleucina-6, GST,* e dos *receptores de progesterona* em mulheres na pós-menopausa com baixa densidade mineral óssea

**DOI:** 10.1590/1516-3180.2014.1321566

**Published:** 2014-02-01

**Authors:** Katia Franco Moura, Mauro Haidar, Claúdio Bonduki, Paulo Cezar Feldner, Ismael Silva, José Maria Soares, Manoel João Girão

**Affiliations:** I Postgraduate Student, Department of Gynecology, Universidade Federal de São Paulo - Escola Paulista de Medicina (Unifesp-EPM), São Paulo, Brazil; II MD, PhD. Adjunct Professor, Department of Gynecology, Universidade Federal de São Paulo - Escola Paulista de Medicina (Unifesp-EPM), São Paulo, Brazil; III MD, PhD. Affiliated Professor, Department of Gynecology, Universidade Federal de São Paulo - Escola Paulista de Medicina (Unifesp-EPM), São Paulo, Brazil; IV MD, PhD. Titular Professor, Department of Gynecology, Universidade Federal de São Paulo - Escola Paulista de Medicina (Unifesp-EPM), São Paulo, Brazil

**Keywords:** Polymorphism, genetic, Interleukins, Receptors, progesterone, Bone density, Postmenopause, Polimorfismo genético, Interleucinas, Receptores de progesterona, Densidade óssea, Pós-menopausa

## Abstract

**CONTEXT AND OBJECTIVE:**

Osteoporosis is a skeletal disorder characterized by low bone mineral density (BMD). Studies have shown that some of the genetic components relating to lower BMD may be detected by polymorphisms. Our aim was to evaluate the frequencies of *interleukin-6, GST *and* progesterone receptor gene* polymorphisms in postmenopausal women with low BMD.

**DESIGN AND SETTING:**

Cross-sectional study, conducted in a public university in São Paulo, Brazil.

**METHODS:**

: We evaluated *interleukin-6 (IL-6), progesterone receptor gene (PROGINS)* and *glutathione S-transferase (GST)* polymorphisms in 110 postmenopausal women with no previous use of hormone therapy. Tests were performed using DNA-PCR, from oral scrapings. We used Student's t-test and a logistic regression model for statistical analysis.

**RESULTS:**

: Regarding *IL-6* polymorphism, 58.2% of the patients were homozygotes (*GG*) and 41.8% had allele *C* (heterozygote or mutant homozygote + *GC or CC). PROGINS* genotype polymorphism was absent in 79% (wild homozygote or P1/P1) and present in 20.9% (heterozygote or P1/P2). Regarding *GSTM1 *polymorphism, the allele (1/1) was present in 72.7% of the patients and was absent in 27.3%. We found that *IL-6* polymorphism had statistically significant correlations with the L2-L4 T-score (P = 0.032) and with BMD (P = 0.005). Women with *IL-6* polymorphism were 2.3 times more likely to have a L2-L4 T-score of less than -1, compared with those not presenting this polymorphism.

**CONCLUSION:**

*IL-6 gene* polymorphism was correlated with low BMD, whereas the *PROGINS* and *GSTM1* polymorphisms did not show any correlation.

## Abstract (pt)

**CONTEXTO E OBJETIVO:**

A osteoporose é uma desordem esquelética caracterizada por baixa densidade mineral óssea. Estudos têm demonstrado que alguns componentes genéticos relacionados com a menor densidade mineral óssea podem ser detectados por polimorfismos. Nosso objetivo foi avaliar a presença do polimorfismo de genes em mulheres pós-menopáusicas com baixa densidade mineral óssea.

**TIPO DE ESTUDO E LOCAL:**

Estudo transversal, conduzido em universidade pública em São Paulo, Brasil.

**MÉTODOS:**

Avaliamos os polimorfismos relacionados à interleucina-6 *(IL-6)*, o gene *receptor de progesterona (PROGINS)* e *glutationa S-transferase (GST)* em 110 mulheres na pós-menopausa sem terapia hormonal prévia. Os testes foram realizados com DNA-PCR a partir de raspados orais. Foram utilizados teste t de Student e modelo de regressão logística para análise estatística.

**RESULTADOS:**

Em relação ao polimorfismo *IL-6*, 58,2% dos pacientes eram homozigotos (*GG*) e 41,8% tinham o alelo C (heterozigoto ou homozigoto mutante + *GC* ou *CC*). Nos genótipos do polimorfismo *PROGINS*, 79% estavam ausentes (homozigoto selvagem ou *P1/P1*) e 20,9% presentes (heterozigoto ou *P1/P2*). No polimorfismo do *GSTM1*, o alelo (1/1) estava presente em 72,7% dos pacientes e ausente em 27,3%. Encontramos significância estatística entre o polimorfismo genético da *IL-6* com o T-*score* de L2-L4 (P = 0,032) e a densidade mineral óssea (P = 0,005). As mulheres com polimorfismo da *IL-6* tiveram 2,3 vezes mais chance de ter menos de -1 na L2-L4 T-*score*, quando comparadas às não portadoras.

**CONCLUSÃO:**

O polimorfismo do gene da *IL-6* está correlacionado com baixa densidade mineral óssea, enquanto os polimorfismos *GSTM1* e *PROGINS* não mostraram correlação.

## INTRODUCTION

Osteoporosis is a skeletal disorder characterized by low bone mineral density (BMD) and deterioration of bone microarchitecture, thus predisposing towards a risk of fractures. It is estimated to affect more than 75 million people worldwide. The risk factors among women include: race, lower height, body mass index, low-calcium diet, use of corticosteroids for over six months, smoking and menopausal status.^1^

Earlier studies showed that environmental effects and genetic control influenced bone turnover.^2^ ^,^ ^3^ Back in 1991, genetic inheritance was shown to be 45-85% likely to be a determinant of mineral density.^2^Since then, various genes have been investigated.^3^ ^-^ ^7^ Studies have shown that some of the genetic components relating to lower BMD may be detected by gene polymorphisms such as *interleukin-6 (IL-6)* polymorphism.^8^ ^-^ ^11^

*IL-6* is a phosphoric acid-containing glycoprotein with 185 amino acids and it is located in chromosome 7p21. It is a multifunctional cytokine produced by mononuclear cells and is regulated by the presence of polymorphisms. In fact, the *C-G* exchange in nucleotide 174 affects the transcription of this gene by decreasing its expression. *IL-6* gene transcriptional activity is also marked in the presence of polymorphism in the promoter region (174 *G/C*). Presence of the *C* allele mutant is associated with low *IL-6* plasma levels; however, presence of the *G* wild allele is related to high plasma levels of this cytosine.^12^ Some authors have observed increased IL-6 levels in postmenopausal women in response to lower levels of estradiol,^13^ and that gene polymorphism influenced bone resorption.^14^

Luo et al.^15^ reported that progesterone stimulated osteoblast proliferation and differentiation. This led to increased growth factors in osteoblasts, thus stimulating their proliferation and extracellular matrix synthesis. *Progesterone receptor (PR)* genes are located in the long arm of chromosome 11 (bands q22-23). Recently, variations in PR genes have been described, such as *PROGINS*. This polymorphism consists of an insertion of the Alu family, with a length of 306 base pairs (bp) in the *G* introns between exons 7 and 8. This event frequently occurs with two other mutations: replacement of a guanine base (G) with thymine (T) in exon 4, thereby exchanging a valine amino acid (Val) for leucine (Leu) in the receptor; and replacement of a cytosine base (C) with thymine (T) in exon 5.^16^ ^,^ ^17^

*Glutathione S-transferases (GSTs)* are a family of enzymes that regulate conversion of toxic compounds to hydrophilic metabolites. *GSTs *are responsible for metabolization and peroxidation of estrogens and lipids, and their polymorphism frequency is related to ethnic factors.^18^ ^,^ ^19^ There are three main genes involved in these polymorphisms: *GSTM1, GSTT1 *and *GSTP1*. *GSTM1 *is a gene located in chromosome 1p13.3 and is not expressed by 20% to 50% of individuals.^18^ Studies have suggested that genetic alterations such as in *GSTM1* may increase the levels of estrogen and/or catechol estrogens, which might be associated with estrogen-dependent diseases.^20^ ^,^ ^21^

## OBJECTIVE

The aim of our work was to estimate the frequencies of *interleukin-6, GST *and *progesterone receptor gene* *(PROGINS)* polymorphisms in postmenopausal women with low BMD. 

## METHODS

This was a cross-sectional study, conducted in a public university in Sгo Paulo, Brazil, including 110 patients with no sample size calculation prior to the study initiation.

One hundred and ten women in their first ten years after the menopause (mean age of 52 years) were recruited. All patients provided their informed consent to participate in this study and the Local Ethics Committee approved the related protocol. This study was supported by the Department of Gynecology and there was no external sponsor. 

The inclusion criteria were that the women needed to have been postmenopausal for 5 to 10 years and needed to present follicle-stimulating hormone, FSH > 35 mUI/ml and estradiol < 20 pg/ml, and that a bone densitometry scan done before hormone therapy needed to be available.

The exclusion criteria comprised absence of bone densitometry before hormone therapy and no use of corticosteroids. None of the subjects had received any medication known to affect bone metabolism (such as glucocorticoids, thyroxin, anti-epileptics, bisphosphonates, calcitonin or hormone replacement therapy for more than three months).

All the subjects underwent careful physical examination and medical history review, including personal data, such as age, race, age at the menopause and medications currently used. Bone mineral density was evaluated using the Lunar DPX-L equipment (Lunar, Madison, Wisconsin, United States), which, according to the criterion of "z-bottom of form, z-top", described by Kiebzak et al.,^22^ achieves a coefficient of variation of 0.62%. The measurements were performed on the lumbar spine. The T-score and the Z-score were calculated. For comparison with polymorphisms, a T-score cutoff at -1 was used to define low bone mineral density. 

Samples of oral scrapings were collected using a cytobrush which was rubbed against the oral mucus lining and then placed in tubes containing tris-ethylenediaminetetraacetic acid (EDTA) buffer solution. The cytological samples thus obtained were preserved at -80 °C until subsequent extraction of the genomic deoxyribonucleic acid (DNA).

DNA extraction was performed in accordance with the Amersham-Pharmacia GFX kit protocol for oral cells. The DNA thus obtained was ready for use in the polymerase chain reaction (PCR). The presence of *IL-6, PROGINS *and* GSTM1* gene polymorphisms was investigated using primers that amplified a small DNA fragment containing the polymorphic site. The primer sequences used for the promoter region of the gene were as follows:

*For interleukin 6: 5ґ - ATG CCA AGT GCT GAG TCA CTA - 3ґ (sense) and 5ґ - GGA AAA TCC CAC ATT TGA TA - 3ґ (antisense). For PROGINS: 5ґ - GGC AGA AAG CAA AAT AAA AAG A - 3ґ (sense) and 5ґ - AAA GTA TTT TCT TGC TAA ATG TC - 3ґ (antisense). For GSTM: 5ґ - GAA CTC CCT GAA AAG CTA AAG C - 3ґ (sense) and 5ґ - GTT GGG CTC AAA TAT ACG GTG G - 3ґ (antisense).*

Demographic and clinical variables were compared between genotype groups for the *IL6, PROGINS *and* GSTM1 *polymorphisms. Statistical analyses were performed using Student's t-test or the Mann-Whitney test. Odds ratios and confidence intervals were derived from binary logistic regression analyses. The significance level was taken to be 0.05. To estimate the risks of the polymorphisms, a logistic regression model was applied to each polymorphism, using a 95% confidence interval.

## RESULTS

In our population, 94% of the women were Caucasian. Regarding *IL-6* polymorphism, the composition of the groups was that 64 women were *GG* (homozygotes) and 48 were *GC/CC* (only two patients were CC). In relation to *PROGINS*, there were 87 women with *P1/P1* (wild homozygotes) and 23 with positive *PROGINS* genotypes of the progesterone receptors, *P1/P2* (heterozygote). The *GSTM1 *polymorphism analysis showed that in 30 women, the allele was absent (0/0) and, in 80 women, the allele genotype was present (1/1). 

Demographic and clinical variables were compared among homozygotes and heterozygotes for the *IL6* genotype. There were significant differences in the L2/L4 T-score (P = 0.03) and BMD (P = 0.05) among the genotype groups (Table 1).

**Table 1:**
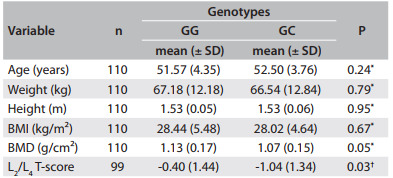
Clinical data regarding the interleukin-6 (IL-6) genotype. Values are given as the mean ± standard deviation (SD)

^*^Student's t; ^†^Mann-Whitney; BMI = body mass index; BMD = bone mineral density

To facilitate the odds ratio calculation, the cutoff point for the L2/L4 T-score was adjusted to -1. In this manner, we found that women with polymorphism in one allele, i.e. who were heterozygous for *IL-6*, presented a risk of having a L2/L4 T-score lower than -1.0 that was 2.3 times higher than those without this polymorphism.

No statistical differences were found between the genotype groups for the *PROGINS and GSTM1* polymorphisms regarding clinical variables (Tables 2 and 3).

**Table 2:**
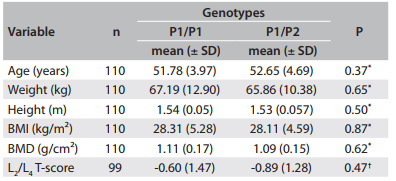
Clinical data regarding PROGINS. Values are given as the mean ± standard deviation (SD)

^*^Student's t; ^†^Mann-Whitney; BMI = body mass index; BMD = bone mineral density

**Table 3:**
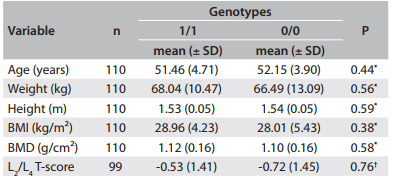
Clinical data regarding the GSTM1 genotype. Values are given as the mean ± standard deviation (SD)

^*^Student's t; ^†^Mann-Whitney; BMI = body mass index; BMD = bone mineral density

## DISCUSSION

In our study, we found that *G-C* polymorphism in region 174 of the *IL-6* gene was associated with low bone mineral density. IL-6 is a cytokine with a crucial role in immune, inflammatory, hematopoietic and atherogenic responses and is associated with bone absorption. In bones, IL-6 is synthesized by osteoblasts, monocytes and T-cells, leading to differentiation and activation of osteoclasts. IL-1 and TNF-alpha have a role in activation, whereas estradiol and glucocorticoids suppress transcription of the *IL-6* gene. Thus, the decreased estrogen levels in postmenopausal women may trigger increased *IL-6* expression, thus leading to bone mass loss.^14^

Several variations in alleles have been identified in the *IL-6* promoter region. A common polymorphism, such as *G-C* exchange at position 174, may also interact with estrogen receptors that regulate *IL-6* expression. There is evidence that this polymorphism produces a functional variant in which the allele *174C* results in low stimulation of *IL-6* and also in low concentrations of IL-6 levels, compared with the presence of the *G* allele.^14^

Czerny et al.^23^ studied associations between cytokine gene polymorphisms (*IL-1 beta, IL-2 *and* IL-6*) and BMD values in postmenopausal women. Their study included 226 postmenopausal women with a diagnosed BMD T-score lower than -2.5 standard deviations (SD) and 224 postmenopausal women with a BMD T-score greater than -2.5 SD. Among the women with T-scores below -2.5 SD, the BMD values were significantly lower in the carriers of the *IL-6 GG* genotype than in those with the *CC* and *GC* genotypes.

In a cohort of 559 postmenopausal Spanish women, two polymorphisms in the *IL-6R* promoter were analyzed in relation to BMD and body mass index. The authors reported that there was a significant association between polymorphisms of the *IL-6R *gene and BMD.^24^

On the other hand, Garnero et al.^25^ found that there were no significant associations between genotypes, bone turnover marker polymorphism and bone turnover or BMD in a cohort of healthy French women. They concluded that *IL-6* polymorphism was weakly associated with the peak BMD level and the rate of postmenopausal forearm trabecular bone loss. According to those authors, *IL-6* genotypes accounted only for a small proportion of the variation of both peak BMD and rate of bone loss.

Even though no specific studies have reported polymorphisms of the *PROGINS* and *GSTM1* genes in relation to BMD, we decided to study these polymorphisms. These occurrences can be explained by progesterone action on bone formation and the influence of *GSTM1* on estrogen metabolization.

It has been implied that *GSTs* are important molecules involved in activation of cytoprotection genes^18^ and in correlation with the breast because of their ability to metabolize estrogens and lipids through peroxidation.^20^ This may also keep osteoporosis from manifesting, due to hyperestrogenism. This change to this gene could influence bone mass.

Some studies have observed that progesterone stimulates proliferation of bone cells. This can be explained by increased insulin growth factor (*IGF-*2) levels, which could potentially stimulate proliferation of osteoblasts.^15^ Growth factor beta, together with insulinoid factor, is the most abundant growth factor in the bone, but the effect of progesterone on growth factor beta in osteoblasts is still unknown.^26^ ^,^ ^27^

In our study, we also evaluated *PROGINS* and *GST* gene polymorphisms regarding age, weight, IMC and BMD variables. We did not find any statistical correlation among them.

We reported that there was a significant association between polymorphisms of the *IL-6* gene and BMD. The clinical importance of these findings may lead to new directions for osteoporosis management, such as biomarkers and molecular targets in therapeutics. 

However, in fact, there were several limitations to our study. It is possible that the sample size was inadequate for detecting small differences in the groups, and that the study power was suboptimal. Moreover, the lack of a matched control group consisting of women with normal BMD limits the conclusions of this study. It is important to reproduce this study in other populations in order to achieve better analysis and functional outcomes.

## CONCLUSION

This study showed that there was a considerable frequency of polymorphisms of the IL-6 gene in women with low BMD. However, this was not found for the other genes under investigation. Knowledge of osteoporosis-related genetic mechanisms may facilitate prevention and selection of women for therapeutics and prognosis.
